# Adult Feeding Preference and Fecundity in the Large Pine Weevil, *Hylobius abietis* (Coleoptera: Curculionidae)

**DOI:** 10.3390/insects12050473

**Published:** 2021-05-19

**Authors:** Petr Doležal, Lenka Kleinová, Markéta Davídková

**Affiliations:** 1 Biology Centre, Czech Academy of Sciences, Institute of Entomology, 37005 České Budějovice, Czech Republic; lenkaperfollova@email.cz (L.K.); davidkova@entu.cas.cz (M.D.); 2 Faculty of Science, The University of South Bohemia in České Budějovice, 37005 České Budějovice, Czech Republic

**Keywords:** pinaceae, fecundity, seedling damage, forest insects, reproduction

## Abstract

**Simple Summary:**

The large pine weevil, *Hylobius abietis*, is a weevil species that causes extensive damage to coniferous seedlings in Eurasia. Its importance in central Europe has recently increased due to sanitation logging and reforestation activities in the areas of recent bark beetle outbreak. *H. abietis* is a polyphagous species and host plants on which pine weevils develop and feed are important determinants of their fecundity and reproductive success. In this study, we tested adult feeding preferences of *H. abietis* in outdoor conditions, and we studied the influence of food source on fecundity. Seedlings of *Pseudotsuga menziesii* were the most preferred, but low oviposition indicated lack of nutrients. Feeding on *Betula pendula* was recorded only in one group of seedlings, and high mortality of weevils was observed. Knowledge of food preferences together with fecundity on alternative food sources may contribute to planning appropriate protective measures and forecasting the damage in areas where former spruce monocultures are being converted to mixed-species stands. Mixed-species plantations could also represent an alternative to insecticidal protection of coniferous seedlings.

**Abstract:**

Adult feeding preferences of *Hylobius abietis* on *Picea abies*, *Larix decidua*, *Pinus sylvestris*, *Pseudotsuga menziesii*, *Abies alba* and *Betula pendula* were tested in outdoor conditions. The preferred food source was *P. menziesii*, and the mean bark area consumed per seedling was 440.8 ± 147.9 mm^2^. The second most preferred host was *P. abies*. The coniferous species that suffered the least damage was *A. alba* (76.8 ± 62.56 mm^2^ per seedling). *B. pendula* was the least preferred source of food, and it caused mortality of 60% of weevils that fed on it. Weevils exhibited large differences in fecundity when fed with different tree species in a laboratory experiment. The largest number of eggs was laid by females fed with *P. abies*. Mean egg numbers reached 26.4 ± 24.89 eggs per experiment for *P. abies*. Similar fecundity was observed in weevils fed with twigs of *P. sylvestris*. Oviposition was approximately six times lower in females fed with *L. decidua* and *P. menziesii.* The maximum number of eggs laid by a single female during a one-month experiment was 90. The results are discussed in relation to management of *H. abietis*.

## 1. Introduction

The large pine weevil *Hylobius abietis* (L.) (Coleoptera: Curculionidae) is an economically important species causing damage in coniferous plantations in Europe and Asia [1,2]. Its pest status is a consequence of modern silvicultural practices that are largely based on clearcuttings and reforestation. Sanitation logging in the areas of recent bark beetle outbreaks in Europe has magnified the amount of available breeding substrate [3,4,5,6]. In the Czech Republic, damage by the large pine weevil has doubled every year since 2014 [7] and *H. abietis* was identified as the major threat to successful reforestation in central and northern Europe [8,9].

Adult pine weevils fly over long distances in the spring to clear-felled areas to find dying stumps and roots of conifers suitable for breeding [10,11]. When such habitat is found, adults feed on twigs and branches within the crown of fully grown trees in the surroundings. After two or three weeks, weevils lose their ability to fly, and feed on the bark of newly planted or naturally regenerated coniferous seedlings for the rest of the season [12,13,14,15]. Feeding typically occurs near the root collar and on the main stem, and damaged seedlings suffer reduced growth or die when the stem is girdled [16,17]. Development of one generation usually lasts one year in central Europe and two years in Scandinavia but can be as long as five years [2,18]. Large pine weevil life cycle depends on local conditions and in the absence of obligatory diapause; temperature is the key factor influencing development, voltinism and abundance. In addition, breeding habitats in roots and stumps eliminate the disadvantage of seasonal food availability and to some extent buffer temperature fluctuations, which may lead to continuous development under suitable conditions [2,9,19,20]. Shortening of development due to warming climate has recently been recorded in central Europe and emergence of a young generation may occur as early as autumn. Consequently, damage in autumn increases after maturation feeding of young beetles [20,21,22]. 

Quality of breeding substrate is another factor that affects both adults and sub-imaginal stages [16,23,24]. *H. abietis* is a polyphagous species [25,26,27]. While adults feed on a variety of coniferous and angiosperm trees [2,24], larval development is limited to a few conifer species, among which pine is preferred over spruce [28,29]. Larvae develop faster and suffer lower mortality rates in pines and spruce than in other conifers [16,30]. Host plants on which pine weevils developed and feed are important determinants of their fecundity and reproductive success. Generally, hosts favored by adults match those of larvae [18,23,30], but feeding has been documented also on *Fraxinus excelsior*, *Alnus glutinosa*, *Fagus sylvatica*, *Quercus robur*, *Salix* spp., *Betula* spp. and other species of broadleaved trees and shrubs. However, some of the above-mentioned species may lack essential nutrients or contain toxic compounds and their consumption reduces damage on conifers and increases weevil mortality [27,31]. From this point of view, establishment of mixed plantings of broad-leaved and coniferous species reduces damages and cost of subsequent protective measures [22,24,29,31].

The objective of this study was to assess the feeding behavior and oviposition rate of *H. abietis* on different plant species. Within this study two experiments were undertaken. The first assessed the adult feeding preferences of *H. abietis* in outdoor conditions when presented with seedlings of five conifer and one broad-leaved species. The second experiment reported here was a laboratory study of the influence of food on fecundity of adults feeding on four species of conifers. The conifers were chosen to reflect the most typical species planted in Central European forestry. Some of these species were tested by previous authors, but for most of them, no studies of *H. abietis* feeding behavior exist. *Betula pendula* was chosen as a supplementary deciduous tree to verify some controversial results regarding the lethal effect of *B. pendula* to pine weevil [24,27,31]. Knowledge on these topics is of high importance for reforestation activities in the areas of recent bark beetle outbreak, where former spruce monocultures are being converted to mixed-species stands. Mixed-species plantations could also represent an alternative to insecticidal protection of coniferous seedlings.

## 2. Materials and Methods

### 2.1. Experimental Insects

Experimental adults were obtained from a laboratory culture maintained on *Picea abies* roots. After emerging, adult beetles were placed in plastic containers in groups of twenty with a tube of water and fed *P. abies* and *Pinus sylvestris* branches. Containers were kept in an insectarium (22 °C, 75% RH, LD 18:6 h). The weevils used in experiments were ca. 90 days old. 

### 2.2. Feeding Preference Experiment

We tested six species of trees—*P. abies*, *Larix decidua*, *P. sylvestris*, *Pseudotsuga menziesii*, *Abies alba* and *Betula pendula*. The plants used in experiments were one year old seedlings obtained from a commercial nursery (Forests of the Czech Republic, state enterprises, Budkov, Czech Republic). Mean height of seedlings ranged from 25 to 30 cm and stem diameter above the soil was ca. 0.5 cm. Seedlings were planted in groups of six in big plastic boxes (30 × 80 × 50 cm^3^; height × length × width). Groups consisted of one seedling of every tree species mentioned above and in total 5 boxes were made and placed in the shaded place in the garden of the Biology Centre in České Budějovice. Wooden frame covered with polypropylene net was then screwed to each of the boxes and ten weevils were released inside in the summer of 2020. Every week, seedlings were inspected and feeding scars on stems measured with transparent millimeter paper. At the end of the experiment, folded trap bark was placed in each box to collect weevils, and soil was inspected for dead beetles. The experiment lasted 6 weeks.

### 2.3. Oviposition Experiment

In this experiment we tested four species of conifers—*P. abies*, *L. decidua*, *P. sylvestris* and *P. menziesii*. One male and one female *H. abietis* were released in a ventilated clear plastic box. Each box contained a twig of one conifer species (length 100 mm, diameter ca. 15 mm) with thin bark serving as a feeding substrate. Prior to the beginning of the experiment beetles were starved for 48 h. A Petri dish with moistened black sand was placed in each box to keep the humidity high and as a substrate for oviposition. In total 40 boxes were prepared, 10 for each tree species. The sex of *H. abietis* was determined according to shape and setae on the last abdominal sternite [32]. The number of laid eggs was recorded at intervals of 2–3 days and twigs replaced with fresh ones. The experiment lasted 28 days. 

### 2.4. Statistical Analysis

Data were analyzed in GraphPad v. 6 (GraphPad Software, San Diego, CA, USA). Repeated measures by row ANOVA were used to assess feeding preference and oviposition on different tree species, the repeated measures factor was time. Tukey’s multiple comparison test was selected to compare the damages recorded on different seedling species tested.

## 3. Results

### 3.1. Feeding Preference Experiment

**Figure 1:**
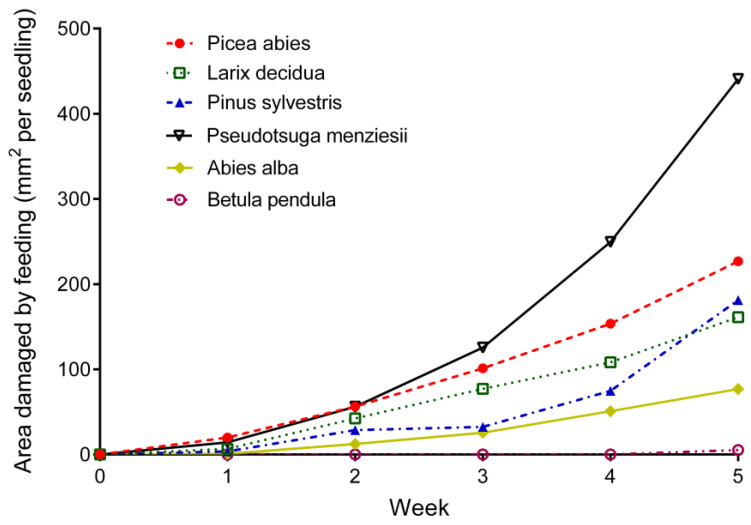
Cumulative mean weekly changes in feeding damage by *Hylobius abietis* adults on seedlings of 6 different tree species.

**Figure 2:**
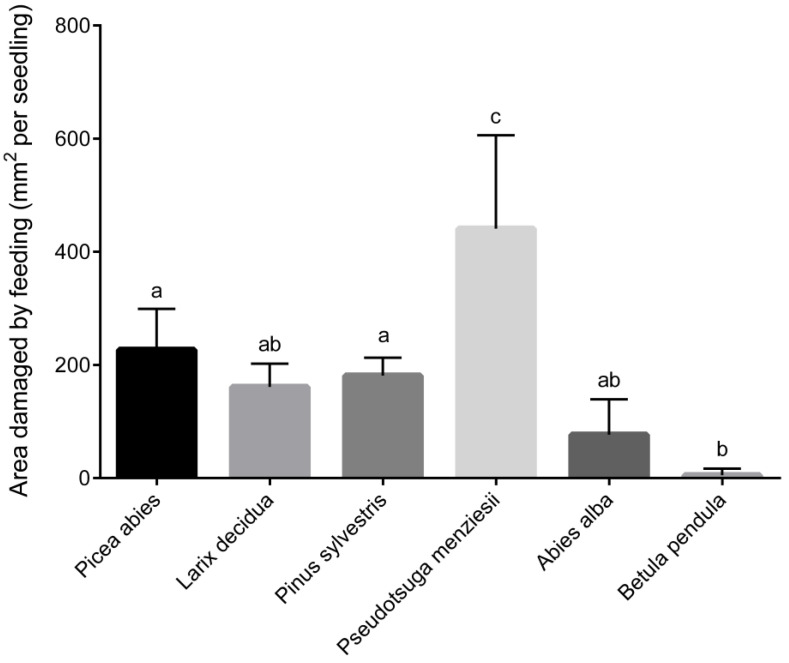
Cumulative mean (± SD) seedling bark surface area consumed at the end of five-weeks experiment on six tree species by *Hylobius abietis* adults. Different letters above the columns indicate differences (*p* < 0.05) (Tukey’s multiple comparison test).

The prevalence of feeding by the weevils differed (RM ANOVA, *p* < 0.0001) among the tree species (Figure 1 and Figure 2). During the first two experimental weeks the preferred food source was *P. abies*. From the third week onwards, weevils started to prefer *P. menziesii* and seedlings of this species sustained the most damage. The mean bark area consumed per seedling was 440.8 ± 147.9 mm^2^. The second most preferred host was *P. abies* (226.8 ± 72.31 mm^2^). For nearly the entire experiment, weevils preferred *L. decidua* over *P. sylvestris*. However, damages on *P. sylvestris* dramatically increased during the last week. At the end of experiment mean debarked area reached 181.2 ± 31.77 mm^2^ on *P. sylvestris* and 161.2 ± 41.10 mm^2^ on *L. decidua*. Coniferous species suffering the least damage was *A. alba*. The mean area consumed per seedling was 76.8 ± 62.56 mm^2^. *B. pendula* was the least preferred source of food. Damage was registered only at the last week of experiment in one cage and mean damaged area reached 5.2 ± 11.63 mm^2^ (Figure 1 and Figure 2). 

All weevils were found at the end of the feeding experiment. The total mortality rate was 16%. However, mortality varied among boxes. The highest mortality of 60% was recorded in the box number 5. In boxes number 2 and 4 mortality reached 10%. No dead weevils were recorded in boxes 1 and 3.

### 3.2. Oviposition Experiment

**Figure 3:**
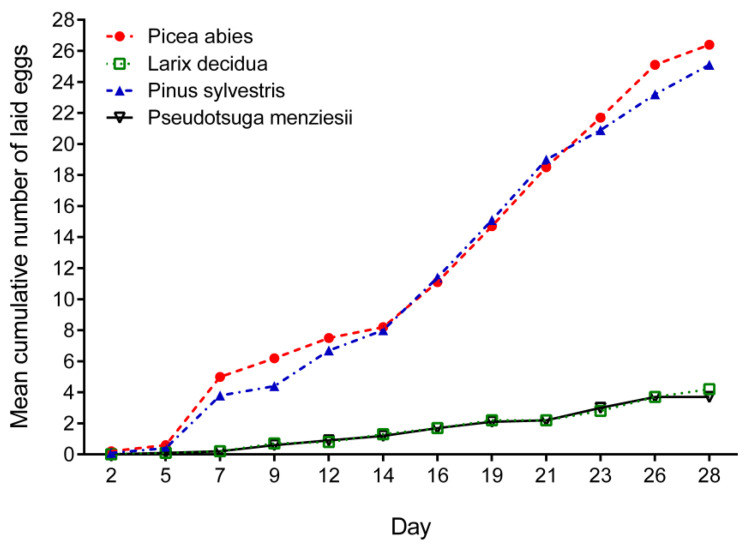
Cumulative number of eggs laid by *Hylobius abietis* females fed with twigs of different tree species. Graph shows average number of eggs laid by one female.

**Figure 4:**
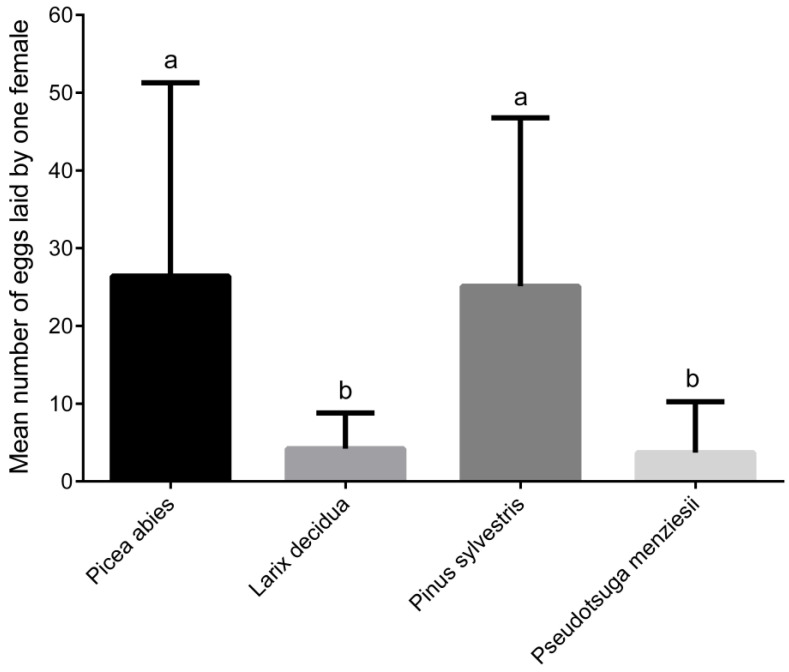
Number of eggs laid by female of *Hylobius abietis* (mean ± SD) during 28 days experimental period. Different letters above the columns indicate differences (*p* < 0.05; RM ANOVA).

Weevils exhibited large differences in fecundity when fed with different tree species. The largest egg numbers were laid by females fed with *P. abies* and *P. sylvestris*. Mean size of egg batches reached 26.4 ± 24.89 eggs per female per experiment in the case of *P. abies*. The maximum number of eggs laid by a single female was 90. Similar fecundity was observed in weevils fed with twigs of *P. sylvestris* (mean 25.10 ± 21.66 eggs per female per experiment, maximum 79 eggs). Females fed with *L. decidua* and *P. menziesii* laid less eggs (RM ANOVA, *p* = 0.0032). Mean size of egg batches per female per experiment was 4.2 ± 4.64 and 3.7 ± 6.55 for these two tree species, respectively. The maximum number of eggs was 13 in females fed with *L. decidua* and 21 in females fed with *P. menziesii* (Figure 3 and Figure 4). 

The average egg production during the entire experiment (6 weeks) was almost similar in females fed with *P. abies* (0.94 eggs per female per day) and *P. sylvestris*, where average number of eggs per female per day reached 0.89. Six times lower values (RM ANOVA, *p* < 0.0001) were recorded in *L. decidua* and *P. menziesii*, 0.15 and 0.13 eggs per female per day, respectively.

## 4. Discussion

Feeding preference experiment results showed conifer tree species are the preferred food source. The largest bark consumption was recorded on *P. menziesii*. These results are in accordance with [33,34] where in both field and laboratory experiments *P. menziesii* and *Picea sitchensis* were the most damaged species. Additionally, relatively high preference for *P. abies*, *P. sylvestris* and *L. decidua* is in agreement with [33]. Similar to our experiment, the only deciduous tree Hybrid aspen (*Populus* × *wettsteinii*) was the least preferred source of food [33]. On the other hand, *P. sylvestris* seems to be the preferred choice in most previously published feeding behavior studies. *P. sylvestris* twigs were preferred over those of *P. abies* in both choice and no choice experiments and weevils consumed twice as much pine bark regardless of temperature regime [29,31]. Results of the present study are contradictory and area of feeding scars on *P. abies* was double than that of *P. sylvestris*, which is consistent with [33]. *P. menziesii* has not been tested in choice experiments by any of the previous authors [29,31] and seedlings of *Larix sp.* are considered less attractive than *P. abies* and *P. sylvestris* [33,35]. Feeding on twigs of *P. sylvestris*, *P. abies*, *B. pendula*, *Acer pseudoplatanus* and *F. excelsior* was tested in no choice experiment [31]. The most preferred source was *P. sylvestris*, but significant damage was recorded also on *B. pendula*, which was preferred over *P. abies* [31]. Although *B. pendula* was the second most eaten species, around 70% of weevils did not feed on it. However, high mortality occurred in beetles that consumed *B. pendula* [31], which is consistent with the results of the present study. The largest debarked area on *B. pendula* was recorded in the box number 5, where mortality exceeded 50%. In contrast, less than 10% of weevils died in other four boxes, where *B. pendula* was not eaten. Previous authors discussed possible effects of thinner bark, lack of nutrients and content of phenolic glycoside compounds as possible explanations for mortality or lower weight gain of weevils feeding on *B. pendula* [27,31]. *A. alba* was the least preferred conifer host. Although the total area of damaged bark was less than 1/6 of those recorded on *P. menziesii*, weevils fed on *A. alba* throughout the whole experiment. 

Food source had a significant effect on the number of eggs laid. The highest fecundity was found in females that consumed twigs of *P. abies* and *P. sylvestris*. Mean egg batches on *P. menziesii* and *L. decidua* reached approximately only a fifth of the two above mentioned species. Although *P. menziesii* was the most damaged host in the feeding preference experiment, oviposition rate indicates its lower nutritional quality, which is consistent with the study [23]. That study reported highest fecundity on *P. abies* followed by *Pinus nigra* and *P. sylvestris*. The size of egg batches on *P. menziesii* was less than half of those on *P. abies*. *P. sitchensis* was the least suitable host with almost ¼ of eggs laid. Daily egg production averages in the present study correspond to the [35,36]. The reported average number of eggs per female per day was 0.8 in weevils fed with *P. sylvestris*. Authors of that study [35] estimated the average female in the first year of her life lays 70 eggs per growth season. This is in accordance with results of the present study. The effect of food source on reproductive behavior can be altered by the female age and the time period that given host species was consumed [36]. Besides food source, other key factors influencing number of laid eggs are female age, oviposition site, and temperature during oviposition period [37,38,39]. Higher oviposition has always been recorded under constant laboratory conditions [37], which may explain the observed maximum numbers of eggs produced by females in our experiments.

The results of current study may have significant implications for reforestation activities in the areas of recent bark beetle outbreak. Among all tested conifer seedlings *P. menziesii* was the most damaged one. Its vulnerability to *H. abietis* feeding should not be neglected since some studies suggest increasing its proportion in newly planted mixtures up to 40% [40]. Therefore, some kind of seedling protection against *H. abietis* should be applied similarly both to *P. abies* and *P. sylvestris*. The results also indicate that some proportion of weevils fed on *B. pendula* even in the presence of coniferous seedlings, which increased their mortality. Planting *B. pendula* together with conifers may therefore reduce the damage by *H. abietis*, and thus the need of insecticidal treatment. Knowledge of food preferences together with fecundity on alternative food sources may contribute to planning appropriate protective measures and forecasting the damages in areas, where former spruce monocultures are being converted to mixed-species stands.

## Data Availability

The data presented in this study are available on request from the corresponding author. The data are not publicly available due to privacy.
